# Comment on: Intracorporeal Versus Extracorporeal Anastomosis in Robotic Right Colectomy: A Multicenter, Triple-Blind, Randomized Clinical Trial

**DOI:** 10.1097/AS9.0000000000000241

**Published:** 2023-02-20

**Authors:** Jeremy Meyer, Rogier Crolla, George van der Schelling

**Affiliations:** From the * Division of Digestive Surgery, University Hospitals of Geneva, Geneva, Switzerland; † Medical School, University of Geneva, Geneva, Switzerland; ‡ Amphia Ziekenhuis, Breda, the Netherlands.

We would like to thank Dohrn et al^1^ for their randomized controlled trial comparing intracorporeal anastomosis (ICA) with specimen extraction through a Pfannenstiel incision with extracorporeal anastomosis (ECA) with specimen extraction through a transverse right upper quadrant incision in patients undergoing robotic right hemicolectomy (rRHC).

The authors state that the main advantage of ICA during rRHC is that it allows for specimen extraction through a Pfannenstiel incision with, as a corollary, reduced postoperative pain and therefore improved postoperative recovery. To this end, they performed a sample size calculation based on the “Quality of Recovery-15” (QoR-15) questionnaire, which is a patient-reported composite outcome. Unfortunately, the trial was not powered on the semiquantitative assessment of postoperative pain or on any objective measurement of postoperative recovery.

Second, the main advantage of the robotic approach for RHC is the improved possibility of performing ICA and choosing a Pfannenstiel incision as the extraction site when compared to laparoscopy. Performing ECA during rRHC offers, to our opinion, absolutely no advantage over ICA, and we therefore question the pertinence of the comparison chosen by the authors, especially when considering the increased cost of the robotic approach. A Pfannenstiel incision is more discreet that a right upper quadrant incision, and is esthetically preferred by patients. On this aspect, we note that the esthetical scoring of the extraction site was not considered in the outcomes and was not included in the QoR-15 questionnaire. Moreover, Pfannenstiel incisions have lower incidence of incisional hernia than transverse right upper quadrant incisions (0.9% vs 3.7%),^2^ which may support ICA over ECA from both a medical point of view but also an economical point of view. This outcome was not assessed in the trial.

Finally, we note that a significant proportion of eligible patients were not randomized and that recruitment was paused during 1 year due to the unavailability of qualified surgeons. However, no threshold of experience was set. We believe it would have been preferable to set a threshold in terms of experience, or to audit the surgical procedures as performed for example in the robotic versus laparoscopic surgery for middle and low rectal cancer trial.^3^ For instance, it was shown that the results of the Robotic-Assisted versus conventional Laparoscopic surgery on risk of conversion to open laparotomy Among patients undergoing Resection for Rectal cancer trial^4^ were potentially biased by the experience of participating surgeons.^5,6^

To conclude, we believe that, despite the results of this randomized controlled trial, ICA should be preferred over ECA during rRHC.

## ACKNOWLEDGMENTS

J.M. conceived and designed the study. All authors interpreted the data and contributed to the writing of the draft manuscript. All authors approved the final version of the manuscript.
